# Proteomic dataset: Profiling of membrane fraction of *Escherichia coli* isolated from Crohn's disease patients after adhesion and invasion experiments

**DOI:** 10.1016/j.dib.2019.104417

**Published:** 2019-08-20

**Authors:** Olga Bukato, Olga Pobeguts, Daria Rakitina, Julia Baikova, Ivan Butenko, Artemy Silantyev, Gleb Fisunov, Vadim Govorun

**Affiliations:** a Federal Research and Clinical Center of Physical-Chemical Medicine of Federal Medical Biological Agency, Russian Federation; b V. P. Serbsky Federal Medical Research Center of Psychiatry and Narcology National Scientific Research Centre on Addictions, Russian Federation

**Keywords:** *Escherichia coli*, AIEC, Crohn's disease, LC-MS/MS, Membrane proteins, Proteome

## Abstract

Crohn's disease (CD) is a type of inflammatory bowel disease (IDB). The endoscopic picture of Crohn's disease includes thickened submucosa, transmural inflammation, fissuring ulceration, and non-caseating granulomas. Intestinal microbiome dysbiosis has been described systematically in patients with IBD. In recent decades it was detailed that *Escherichia coli*, especially adherent-invasive *E. coli* (AIEC) pathotype, has been implicated in the pathogenesis of IBD, including Crohn's disease (Palmela, et al., 2018). In comparison with commensal strains of *E. coli*, AIEC strains have a large adhesive-invasive potential therefore its surface composition is of great interest. We presented a dataset of the membrane proteins of strains isolated from patients with Crohn's disease. From the set of *Escherichia coli* isolated from Crohn's disease patients [2] we chose three isolates with strongest AIEC pathotype. We performed proteome-wide LC-MS analysis of membrane fraction of this isolates after invasion or adhesion-invasion to human intestinal CaCo-2 cell line and prior to this (control). The data including LC-MS/MS raw files and exported MaxQuant search results with fasta files were deposited to the PRIDE repository project accession PXD014250.

**Table:** Specifications Table

Subject	Biology
Specific subject area	Proteomics
Type of data	LC-MS/MS data and identification data
How data were acquired	microflow HPLC system Eksigent Ekspert (Sciex, USA) with autosampler Eksigent Ekspert 400 (Sciex, USA) coupled to ABSciex 6600 with a microflow AB Sciex Duo Spray Ion Source (Sciex, USA)
Data format	Raw and analyzed data
Parameters for data collection	We analyzed membrane fractions of clinical isolates of *E.coli* after invasion or adhesion-invasion to human intestinal CaCo-2 cell line and prior to this (control).
Description of data collection	Dataset covers 54 samples (three biological replicates and two technical).
Data source location	Research and Clinical Center of Physical-Chemical Medicine, Moscow, Russian Federation
Data accessibility	The mass spectrometry proteomics data have been deposited to the ProteomeXchange Consortium via the PRIDE [3] partner repository with the dataset identifier PXD014250 https://www.ebi.ac.uk/pride/archive/projects/PXD014250
Related research article	Bukato O, Garanina I, Matyshkina D, Pobeguts, O, Rakitina D, Baykova J, Ladygina V, Scherbakov P, Govorun V. (2017) Proteomic profiling of *E. coli*, isolated from Crohn's disease patients. FEBS JOURNAL, 284: SpT.5.3001, https://doi.org/10.1111/febs.141.

**Table:** 

**Value of the Data** • The dataset contains the first published wide-range proteome analysis of membrane fraction of *Escherichia coli* isolates from Crohn's disease patients after invasion or after adhesion-invasion to CaCo-2 cell line and prior this. • The data are useful in studies of Crohn's disease or IBD pathogenesis. • These data are valuable for researchers interested in *E. coli* proteomics. • The data contain three biological and two technical replicates of E. coli culture that allows for extended statistical analysis.

## 1 Data

In the microbiome of patients with CD there is an increase in certain members of Enterobacteriaceae, such as *Escherichia coli* [1]. So three strains were isolated from CD patients [2]. We analyzed *E.coli* in three conditions: before invasion or adhesion-invasion (control), after adhesion-invasion or after invasion to CaCo-2 cell line. Membrane fractions were isolated and proteins were assessed in an untargeted label-free bottom-up proteomic experiment using IDA approach (i.e. Information Dependent Acquisition) on AB Sciex TripleTOF 6600 Q-TOF mass-spectrometer coupled with LFQ (label-free quantification) by MaxQuant software. Dataset covers 54 samples (three biological replicates and two technical).(see Table 1)

**Table 1:** Sample description.

Sample name	Strain	Description	Biological replicate	Technical replicate
OB0170_20181203_1	K5	before adhesion and adhesion-invasion	1	1
OB0170_20181203_2	K5	before adhesion and adhesion-invasion	1	2
OB0171_20181203_1	K5	before adhesion and adhesion-invasion	2	1
OB0171_20181203_2	K5	before adhesion and adhesion-invasion	2	2
OB0172_20181203_1	K5	before adhesion and adhesion-invasion	3	1
OB0172_20181203_2	K5	before adhesion and adhesion-invasion	3	2
OB0173_20181203_1	K5	after adhesion-invasion	1	1
OB0173_20181203_2	K5	after adhesion-invasion	1	2
OB0174_20181203_1	K5	after adhesion-invasion	2	1
OB0174_20181203_2	K5	after adhesion-invasion	2	2
OB0175_20181203_1	K5	after adhesion-invasion	3	1
OB0175_20181203_2	K5	after adhesion-invasion	3	2
OB0176_20181203_1	K5	after invasion	1	1
OB0176_20181203_2	K5	after invasion	1	2
OB0177_20181203_1	K5	after invasion	2	1
OB0177_20181203_2	K5	after invasion	2	2
OB0178_20181203_1	K5	after invasion	3	1
OB0178_20181203_2	K5	after invasion	3	2
OB0179_20181203_1	BruB2	before adhesion and adhesion-invasion	1	1
OB0179_20181203_2	BruB2	before adhesion and adhesion-invasion	1	2
OB0180_20181203_1	BruB2	before adhesion and adhesion-invasion	2	1
OB0180_20181203_2	BruB2	before adhesion and adhesion-invasion	2	2
OB0181_20181203_1	BruB2	before adhesion and adhesion-invasion	3	1
OB0181_20181203_2	BruB2	before adhesion and adhesion-invasion	3	2
OB0182_20181203_1	BruB2	after adhesion-invasion	1	1
OB0182_20181203_2	BruB2	after adhesion-invasion	1	2
OB0183_20181203_1	BruB2	after adhesion-invasion	2	1
OB0183_20181203_2	BruB2	after adhesion-invasion	2	2
OB0184_20181203_1	BruB2	after adhesion-invasion	3	1
OB0184_20181203_2	BruB2	after adhesion-invasion	3	2
OB0185_20181203_1	BruB2	after invasion	1	1
OB0185_20181203_2	BruB2	after invasion	1	2
OB0186_20181203_1	BruB2	after invasion	2	1
OB0186_20181203_2	BruB2	after invasion	2	2
OB0187_20181203_1	BruB2	after invasion	3	1
OB0187_20181203_2	BruB2	after invasion	3	2
OB0188_20181203_1	ZvL	before adhesion and adhesion-invasion	1	1
OB0188_20181203_2	ZvL	before adhesion and adhesion-invasion	1	2
OB0189_20181203_1	ZvL	before adhesion and adhesion-invasion	2	1
OB0189_20181203_2	ZvL	before adhesion and adhesion-invasion	2	2
OB0190_20181203_1	ZvL	before adhesion and adhesion-invasion	3	1
OB0190_20181203_2	ZvL	before adhesion and adhesion-invasion	3	2
OB0191_20181203_1	ZvL	after adhesion-invasion	1	1
OB0191_20181203_2	ZvL	after adhesion-invasion	1	2
OB0192_20181203_1	ZvL	after adhesion-invasion	2	1
OB0192_20181203_2	ZvL	after adhesion-invasion	2	2
OB0193_20181203_1	ZvL	after adhesion-invasion	3	1
OB0193_20181203_2	ZvL	after adhesion-invasion	3	2
OB0194_20181203_1	ZvL	after invasion	1	1
OB0194_20181203_2	ZvL	after invasion	1	2
OB0195_20181203_1	ZvL	after invasion	2	1
OB0195_20181203_2	ZvL	after invasion	2	2
OB0196_20181203_1	ZvL	after invasion	3	1
OB0196_20181203_2	ZvL	after invasion	3	2

## 2 Experimental design, materials, and methods

### 2.1 Bacterial strains and cell line

*Escherichia coli* isolates from Crohn's disease patients were used in this experiment. Strains ZvL, BruB2 and K5 isolated from aspirate from ileum lumen, ileum biopsy or feces of CD-patients with the confirmed disease, correspondingly [2]. Overnight culture of cells was diluted 50 times and was grown at 37°C with shaking in Lysogeny Broth (LB) until mid-log phase. The cells were collected by centrifugation (5000 g for 5 min), resuspended in sterile PBS buffer, and then diluted in the same buffer to OD600 = 0.2 (xMark™ Microplate Absorbance Spectrophotometer, Bio-Rad, USA). The bacterial cells (150 μL) were diluted in 5 mL Dulbecco's Modified Eagle medium (DMEM) supplemented with 20% fetal bovine serum (FBS), and 500 μL of this mixture was added to a monolayer of CaCo-2 cell line 1:10 (CaCo-2:*E. coli*). The co-culture was maintained in DMEM with 20% FBS for 3 h at 37^°^C.

### 2.2 Preparation of adherent-invasive *E. coli*

A monolayer of infected CaCo-2 cells was washed twice in sterile PBS buffer pH 8.0. After that, 200 μL of 0,05% Trypsin solution in sterile PBS was added to the monolayer followed incubation for 15 min at room temperature (RT). Then CaCo-2 cells were added to 200 μL of DMEM with 20% FBS and 200 μL of 1% Triton X100 with follow dilution 10^−2^ and 10^−3^.50 μL of each dilution was transferred to a Petri dish with LB agar and incubated overnight at 37^°^C.

### 2.3 Preparation of invasive *E. coli*

A monolayer of infected CaCo-2 cells was washed twice with sterile PBS buffer pH 8.0. After the addition of 1 mL DMEM with 20% FBS containing 300 μg/mL gentamicin to kill extracellular *E.coli* for 1h at 37^°^C, sterile PBS buffer washing twice and 0,05% trypsin solution treatment, cells were diluted with 200 μL DMEM +20% FBS and disrupted with 200 μL 1% Triton X100 without dilution. 50 μL of cells were plated onto LB agar, incubated overnight at 37^°^C.

### 2.4 Isolation of the membrane fraction

Extraction of proteins of the outer membranes of *E. coli* was performed using the carbonate method [4]. The individual colonies from LB agar dish after adhesion-invasion or invasion were grown at 37°C with shaking in LB to log-phase. As a control, *E.coli* cells before co-cultivation with CaCo-2 were used. Cells were collected by centrifugation (5000 g for 5 min). The pellet was washed once with PBS buffer and resuspended in 200 μl of 50 mM Tris-HCl pH 7.5 with the addition of 1 μl of a mixture of nucleases (GE Healthcare). Cells were lysed by ultrasonication for 1 minute at 4°C, clarified by centrifugation (5000 g for 5 min). 1.5 ml of 100 mM Na2CO3 (Sigma) was added to the supernatant and incubated on ice with stirring for 1 h. The membrane fraction was precipitated by ultracentrifugation (115000 g for 30 min), the precipitate was washed with 50 mM Tris-HCl pH 7.5 and collected again at the same speed.

### 2.5 Tryptic digestion

Protein trypsinolysis was performed as described in Ref. [5] with some alterations. Membrane pellets were washed with PBS buffer and resuspended in 15 μl of 10% sodium deoxycholate (DCNa) with the addition of 1 μl nuclease mix (Promega). The suspension was incubated for 1 h at +4 °C. Then the sample was diluted in 100 μl of 100 mM Tris-HCl pH 8.0, 2.5 mM EDTA, and incubated for 30 min at RT. After centrifugation at 14 000 g for 10 min, protein concentration was measured in supernatant by Bradford assay (Quick Start Bradford Protein Assay, BioRad) and samples were equalized. Reducing agent TCEP was added to the supernatant to a final concentration 5 mM, incubated for 1 h at 37°C. Then iodoacetamide was added to a final concentration 30 mM, incubated for 30 min at RT in the dark. Finally, for neutralization the remaining iodoacetamide we added another half of TCEP, incubated for 20 min at RT. After that, samples were diluted with 6x volumes of 100 mM Tris-HCl pH 8.0 and protein hydrolysis was performed by addition of trypsin (in ratio trypsin: protein equal 1: 50, Trypsin Gold, Mass Spectrometry Grade (Promega)) in 0.1% DCNa and incubation at 37°C for 17 h. At this point, trypsinolysis stopped by addition of 50% trifluoroacetic acid (TFA), pH after TFA addition should be 2.0. After centrifugation for 15 min at 14 000 g, the supernatant was collected and cleaned with cartridges C18 (Discovery DSC-18 Tube (Supelco)) according to the manufacturer's protocol. Achieved peptide extracts were dried at SpeedVac (Labconco) and dissolved in 15 μl of LC-MS/MS sample buffer containing 3% acetonitrile and 0.1% TFA. The equivalent of 5 μg of protein was loaded onto HPLC-MS/MS analysis [6].

### 2.6 LC-MS analysis

The LC-MS/MS analysis of the tryptic peptides was performed using a microflow HPLC system Eksigent Ekspert (Sciex, USA) with autosampler Eksigent Ekspert 400 (Sciex, USA) coupled to AB Sciex Triple Tof 6600 with a microflow AB Sciex Duo Spray Ion Source (Sciex, USA). The chromatographic separation of the peptides was performed with Eksigent Column, 2.7 μm, HALO Fused-Core C18, 50 × 0.5 mm 805–10100. Chromatographic separation was performed with the following parameters: a flow rate of 5 μL/min: a 12-min linear gradient of acetonitrile in water with 1% methanol and 0.1% formic acid. The ion source settings were as follows: the flow rate of the nebulizer gas - 10 L/min, the voltage applied to the capillary - 4000 V. The mode of obtaining MS spectra is as follows: accumulation time 50 ms, mass/charge ratio range from 100 to 2500 *m*/*z*, criteria for preferred selection of ions for isolation and fragmentation - charge 2–5, the number parent ions from one MS spectrum - 25, the minimum intensity is 5000. After the first analysis, the ion was excluded from candidates for fragmentation for 5 s. The fragmentation parameters were as follows: the accumulation time of the spectra fragmentation - 20 ms, mass/charge ratio range from 100 to 2000 *m*/*z*, full cycle time is 600 ms.

### 2.7 Protein identification and quantitative analysis

Identification and label-free quantification analysis were performed with MaxQuant software with default settings. Each CD-isolated strain was calculated against a database of proteins created directly for this strain. These databases were formed on the basis of genome of strain isolated from a patient with Crohn's disease [2]. All genes sequences were translated and annotated with Prokka software and Human keratins were added to all databases to avoid misinterpretation of contaminating proteins.
